# Immunization of Epidemics in Multiplex Networks

**DOI:** 10.1371/journal.pone.0112018

**Published:** 2014-11-17

**Authors:** Dawei Zhao, Lianhai Wang, Shudong Li, Zhen Wang, Lin Wang, Bo Gao

**Affiliations:** 1 Shandong Provincial Key Laboratory of Computer Network, Shandong Computer Science Center (National Supercomputer Center in Jinan), Jinan, China; 2 College of Mathematics and Information Science, Shandong Institute of Business and Technology, Shandong, Yantai, China; 3 College of Computer, National University of Defense Technology, Hunan, Changsha, China; 4 Department of Physics, Hong Kong Baptist University, Kowloon Tong, Hong Kong; 5 Center for Nonlinear Studies and Beijing-Hong Kong-Singapore Joint Center for Nonlinear and Complex systems (Hong Kong), Institute of Computational and Theoretical Studies, Hong Kong Baptist University, Kowloon Tong, Hong Kong; 6 Centre for Chaos and Complex Networks, Department of Electronic Engineering, City University of Hong Kong, Hong Kong; 7 School of Computer Information management, Inner Mongolia University of Finance and Economics, Hohhot, China; Beijing University of Posts and Telecommunications, China

## Abstract

Up to now, immunization of disease propagation has attracted great attention in both theoretical and experimental researches. However, vast majority of existing achievements are limited to the simple assumption of single layer networked population, which seems obviously inconsistent with recent development of complex network theory: each node could possess multiple roles in different topology connections. Inspired by this fact, we here propose the immunization strategies on multiplex networks, including multiplex node-based random (targeted) immunization and layer node-based random (targeted) immunization. With the theory of generating function, theoretical analysis is developed to calculate the immunization threshold, which is regarded as the most critical index for the effectiveness of addressed immunization strategies. Interestingly, both types of random immunization strategies show more efficiency in controlling disease spreading on multiplex Erdös-Rényi (ER) random networks; while targeted immunization strategies provide better protection on multiplex scale-free (SF) networks.

## Introduction

The structure and dynamics of multiplex networks have attracted much attention by the scientific communities [1]–[17]. Composed of a set of networks integrated by interconnected layers, the multiplex networks well describe many real-world complex systems, such as social networks, communication networks and transportation networks (see [1] for a recent review).

Recently, an increasing number of works has tried to understand the dynamics of epidemic spreading in multiplex networks. Along this line, various mechanisms aiming at exploring the disease propagation process in multiplex topology are proposed and investigated. Examples include competing epidemics [18], the effect of the interconnected network structure [19], joint spreading of both information and disease [20], [21], mutual interaction of both social and epidemic spreading [22], the impact of network correlation patterns [11], to name but a few. Looking at some examples more specifically, in a recent research [10], where epidemic can only spread on partially overlapped networks, the authors reveal that the epidemic threshold would decrease monotonically with the increment of overlapped fraction. Considering the SIR compartmental epidemic model in a multiplex network composed of a virtual layer and a physical layer [11], Yagan *et al.* unfolds the prevalence of the disease in both layers, even if the epidemiological parameters are assigned values lower than the epidemic threshold of each layer. By superposition processing of the network layers, Zhao *et al.* show that a strong positive degree-degree correlation of nodes in different layers could lead to a clearly low epidemic threshold and a relatively smaller infection size [12]. Interestingly, these measures are not significantly affected by the average similarity of neighbors.

As above described, though there have been some achievements focusing on the effect of multiplex architecture on the epidemic dynamics and the resulting threshold, the impact of such increased complexity on the immunization strategies is still virgin [23]–[26]. In the traditional study of network immunization, the vaccinated candidate nodes are usually selected randomly, or chosen intuitively according to their topological properties such as degree, betweenness or 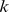-shell, etc [27]–[31]. Thus, an interesting question naturally poses itself, which we aim to address in what follows. If we consider the basic immunization cases in multiplex networks, how do them affect the disease propagation?

Here, with the SIR epidemic model on multiplex networks [12], we explore the performance of several typical immunization strategies, including multiplex node-based random (targeted) immunization and layer node-based random (targeted) immunization. Based on the theory of generating function, mathematical analysis is utilized to distinguish the critical immunization threshold. Extensive computational simulations are used to verify our analysis. We reveal that the efficiency of proposed immunization strategies rely on topology details of multiplex networks.

### Immunization Strategies

For simplicity (yet without loss of generality), we consider the SIR dynamics as the epidemiology model, and then inspect the effect of immunization strategies on disease propagation in multiplex networks. With regard to networks, we select multiplex Erdös-Rényi (ER) random networks [32] and Barabási-Albert scale-free (SF) networks [33]. For such a multiplex framework, it is composed of 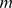 network layers, each of which contains 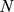 nodes (namely, each node has the replica in different layers). At each time step, every node can fall into one of three states: susceptible (S), infected (I), or recovered (R). On each network layer 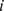, the infected node can infect its susceptible neighbors with transmissibility probability 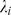; and the infected node is also able to the recovery with probability 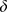. To be simple, we use the case of 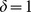.

For networked immunization, there is usually one critical index, immunization threshold 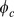 (namely, the required minimum fraction of immunized nodes) [23]–[25], which elevates the efficiency of immunization strategies. Above this threshold, the number of infected nodes is null. Up to now, immunization strategies of single network have been numerously proposed to lower the value of 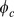 [26]–[31]. However, different from the single network, each node of multiplex networks has a replica in each network layer. To distinguish the node of multiplex networks and its replica in each network layer, we define the terminology: multiplex node and layer node, respectively. Naturally, immunization of multiplex networks can be classified into multiplex node-based immunization and layer node-based immunization. It is worth mentioning the difference of two immunization scenarios: the former means that all the replicas of the same node take immunization, while the latter could just provide protection for one replica in the certain network layer. In what follows, we will investigate the multiplex node-based and layer node-based immunization strategies in multiplex networks, and provide theoretical frame to calculate the critical immunization threshold of different immunization strategies.

### Multiplex node-based immunization

Multiplex node-based immunization refers to the case that a fraction of multiplex nodes is random or targeted immunized. If we use 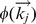, where 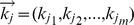 is the degree of a multiplex node 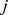 in each layer, to denote the probability that a multiplex node with degree 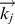 is immunized, then the generating function [34] of the joint degree distribution, for multiplex node-based immunization, could be defined as
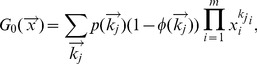
(1)


where 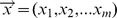 is used to denote the auxiliary variables coupled to 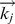 and 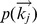 indicates the probability that a randomly chosen multiplex node has degree 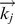. The generating function of remaining joint degree distribution by following a randomly chosen link of network layer 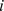 is given by
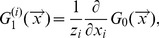
(2)


where 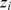 is the average degree of network layer 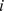.

Then, the probability 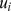 (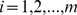) that a multiplex node connects to a link of the chosen network layer 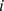 and belongs to the infected cluster is given by the coupled self-consistency equations
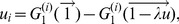
(3)


where 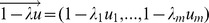.

Thus, the existence of epidemic regime under multiplex node-based immunization requires the largest eigenvalue 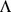 of the Jacobian matrix of Eq. (3) at (0,0,…,0) to be larger than unity [11], [12]. For multiplex networks formed by two network layers (duplex networks), 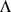 can be expressed as
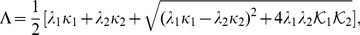
(4)


where
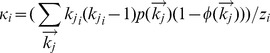



and
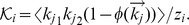



#### Multiplex node-based random immunization

For multiplex node-based random immunization, each node has the same probability to be immunized, so we can write 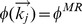 for 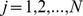. Furthermore, the critical immunization threshold 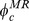 will be the value of 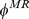 which satisfies 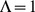 in Eq. (4).

#### Multiplex node-based targeted immunization

For multiplex node-based targeted immunization, the nodes are generally immunized according to their degree, betweeness or 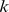-shell, etc. However, since the transmissibility of epidemics in each layer may be different, the role of multiplex nodes may be not identical (even if they have the same degree 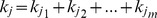). Thus, we define a new index, spreading degree 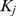, which takes the transmissibility of epidemics into account, to evaluate the importance of multiplex nodes as follows
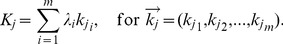
(5)


Under this immunization framework, the immunized probability of a multiplex node with spreading degree 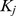 could be expressed as
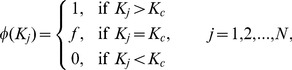
(6)


where 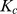 is the cutoff spreading degree for immunization, and 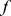 is the immunized probability of nodes with spreading degree 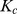. Consequently, the total fraction of immunized nodes is given by
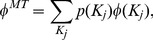
(7)


where 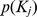 indicates the fraction of nodes with spreading degree 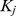. Thus, in the case of multiplex node-based targeted immunization, the critical immunization threshold 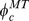 will be the value of 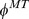 satisfying 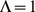 when 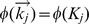 in Eq. (4).

**Figure 1:**
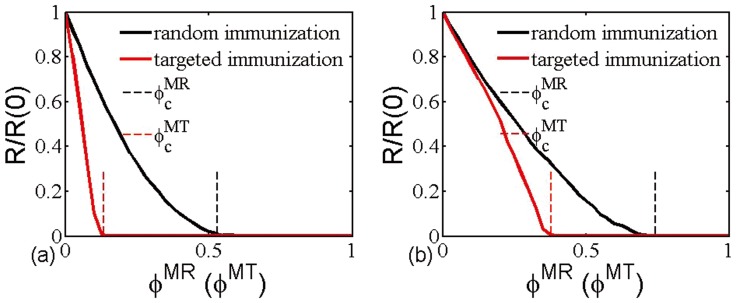
Relative size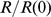 of infected clusters versus the fraction 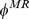 (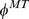) of immunized nodes for multiplex node-based random or targeted immunization. The dash lines denote theoretical immunization thresholds 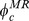 and 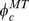. The networks used are multiplex ER networks with average degree (a) 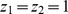, (b) 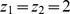; number of layers 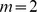 and the size 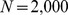. In all the figs, we use the value of transmission rate 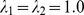.

**Figure 2:**
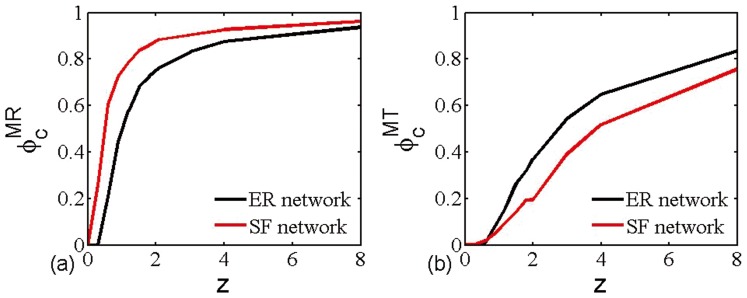
Theoretical immunization thresholds (a)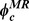 and (b) 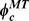 versus average degree 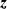 in multiplex ER and SF networks. Networks have the same average degree 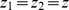 (*i.e.* 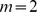) and the size of networks is 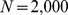.

Fig. 1 shows the theoretical immunization thresholds 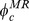 and 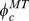 (vertical dash lines) of multiplex node-based random and targeted immunization on the multiplex ER networks with different average degree. Besides, we also show how the relative size 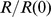 of infected clusters varies in dependence on the fraction 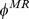 (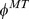) of immunized multiplex nodes, where 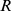 denotes the size of infected clusters and 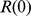 is the value of 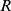 when no node takes immunization. It is clear that the theoretical immunization thresholds are accurate in evaluating the existence of epidemic regime, irrespective of immunization strategies and average degree of networks. More interestingly, random immunization strategy shows larger threshold than that of targeted immunization, which means that the complete eradication of infection risk needs more chosen nodes to take immunization under the framework of random immunization. To compare the efficiency of multiplex node-based immunization strategies, we also introduce them into multiplex SF networks. As shown in Fig. 2(a), the critical immunization threshold 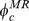 of multiplex ER networks is always lower than that of multiplex SF networks in the case of random immunization. This means multiplex node-based random immunization is more efficiency in multiplex ER networks. At variance, multiplex node-based targeted immunization can provide better protection in multiplex SF networks (see Fig. 2(b)).

### Layer node-based immunization

Besides above proposal, the objects of immunization now turn to layer nodes. If we define 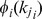 as the probability that a layer node with degree 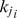 is immunized, then the generating function of the joint degree distribution could be expressed as
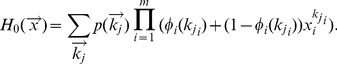
(8)


The generating function of remaining joint degree distribution by following a randomly chosen link of network layer 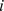 will be
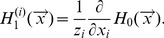
(9)


For layer node-based immunization, the probability 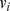 (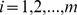) that a multiplex node connects to the chosen network layer 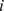 and belongs to the infected cluster is given by
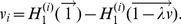
(10)


Similarly, the existence of epidemic regime requires the largest eigenvalue 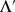 of the Jacobian matrix of Eq. (10) at (0,0,…,0) to be larger than unity. For multiplex networks, 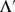 can be expressed as
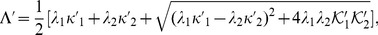
(11)


where
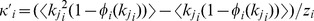



and
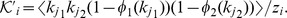



#### Layer node-based random immunization

For layer node-based random immunization, each node in the same network layer has equal probability of taking immunization. So, we can get 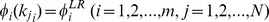. For duplex networks, the critical immunization threshold can be calculated via
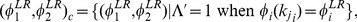
(12)


where 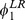 and 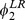 are the fraction of immunized nodes on both network layers, respectively.

#### Layer node-based targeted immunization

For the layer node-based targeted immunization, the immunized probability of a layer node with degree 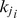 is determined by its degree as follows
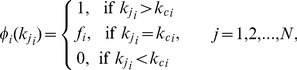
(13)


where 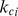 is the cutoff degree for immunization in layer 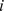, and 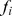 is the immunized probability of nodes with degree 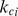. Consequently, the total fraction of immunized nodes in network layer 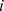 is given by
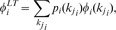
(14)


where 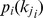 indicates the fraction of nodes with degree 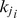 in network layer 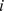.

Thus, for layer node-based targeted immunization of duplex networks, the critical immunization threshold is given by
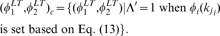
(15)


**Figure 3:**
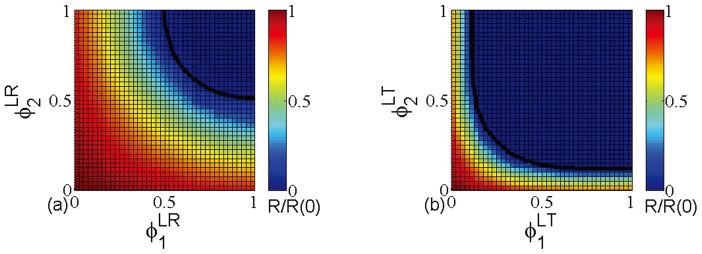
The phase diagram for relative size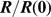 of infected clusters for (a) layer node-based random immunization; (b) layer node-based targeted immunization on multiplex ER networks. The black line indicates the theoretical immunization threshold of both immunization strategies. The networks have the average degree 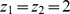 (*i.e.* 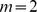), size 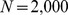.

In Fig. 3, we present the color code of relative size 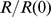 of infected clusters, and use the black line to indicate the theoretical immunization thresholds of layer node-based random [Fig. 3(a)] and targeted immunization [Fig. 3(b)]. Together with the results of Fig. 1, we could prove that the proposed theoretical framework allows us to accurately calculate the immunization thresholds of multiplex networks under different immunization strategies. Moreover, we also notice that layer node-based targeted immunization can eradicate the disease even with lower fraction of immunized nodes in multiplex ER networks, which is similar to the observation of Fig. 1.

**Figure 4:**
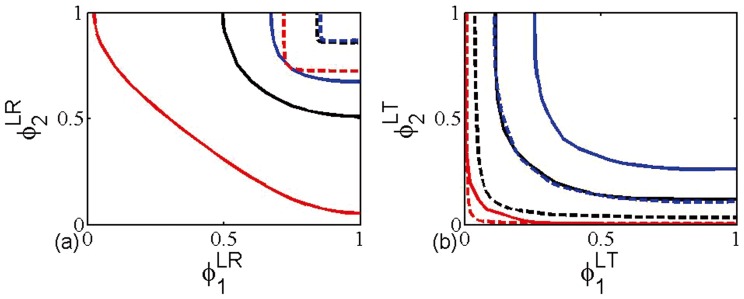
Theoretical immunization threshold for (a) layer node-based random immunization and (b) layer node-based targeted immunization. The networks used are multiplex ER networks (solid line) and multiplex SF networks (dashed line) with average degree 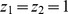 (red), 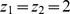 (black), and 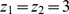 (blue). The size of networks is 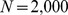.

Subsequently, we further extend the layer node-based immunization proposals to multiplex SF networks and compare its efficiency with case of multiplex ER networks in Fig. 4. It is interesting that we find that the layer node-based random immunization of multiplex ER networks is more effective than that of multiplex SF networks. However in the case of layer node-based targeted immunization, the efficiency of multiplex SF networks is better. Combining with foregoing results, we can get that random immunization is better in multiplex ER networks and targeted has higher efficiency in multiplex SF networks, irrespective of multiplex node or layer node.

## Conclusion

To sum, we propose four kinds of immunization strategies of multiplex networks, including multiplex node-based random immunization and targeted immunization, and layer node-based random immunization and targeted immunization. By using generating function methods, we provide one new theoretical framework which allows us to accurately calculate the critical immunization thresholds of different immunization strategies. We also evaluate the efficiency of the proposed immunization strategies for multiplex ER networks and multiplex SF networks. We show that both multiplex node-based and layer node-based random immunization has higher efficiency in multiplex ER networks, while two types of targeted immunization strategies can provide better protection in multiplex SF networks.
